# The Matron's Books

**Published:** 1907-07-27

**Authors:** 


### Nursing Administration.

### THE MATRON'S BOOKS.

### III.—REGISTER OF SISTERS AND NURSES.

In previous articles specimens were given of the registers in use at some of the leading hospitals, and a form was suggested embodying the best features of those quoted.

But the record should not cease when the probationer's years have run out. The series of registers to be complete must include one continuing the

Register of Sisters and Nurses.

*Name*............... *Age*.............. *Folio*..............

| Date | Appointment | Remarks |
|---|---|---|
| | | |

Date of leaving ..........................................................
Appointment (or cause of leaving)....................................
Subsequent nursing career ...............................................

tale in the event of the nurse being appointed to a post on the regular staff. The register of sisters and nurses records the changes which take place in the distribution of the ward staff, and though less detail is necessary than in the case of learners, there should at the least be entries of the dates when new posts are entered upon, and occasional remarks when called for on the character of the work accomplished under any special circumstances.

In hospitals where the sisters are promoted from the staff, the folio relating to past experience being indicated above, this form suffices, but in the event of outside sisters being appointed, space must be left for their record before joining the staff.

It may be imagined that a distressing amount of labour is entailed upon the matron by these careful and minute records, of what are, after all, viewed from outside but small events. That Nurse C. has taken the place of Sister H. during the latter's absence from influenza may not appear a matter of great moment, but when the next sister's post falls vacant Nurse C. has all the better chance of obtaining it, if instead of a vague memory of her having acted for a short time as stop gap, the matron has a clear record of her having filled the post creditably for five weeks under circumstances of unusual pressure. There are doubtless matrons who never depend, or need to depend, on written entries. Their memory is unerring, and acts automatically. Yet such gifted matrons would do well to remember that the official who trusts exclusively to memory is preparing a difficult path for her successor to tread, and that at any minute unforeseen absence may plunge the whole organisation into confusion for want of the records which the forgetful people are compelled to rely on.

Filling in the registers is not a thing to be done hurriedly day by day. It needs a little quiet time free from interruption, and it is necessary if no details are to be forgotten, that a rough scheme of the constant changes which take place in the disposition of the nursing staff shall be kept up, from which the registers can be compiled. In a large hospital, liable to frequent changes, the following sheet will be found a convenient form for use as a rough diary, to be filled in by the assistant matron once a week,

Week Beginning......................

| Ward | Day | | | | Night | | — |
|---|---|---|---|---|---|---|---|
| | Sister | Staff Nurse | Senior Probrs. | Junior Probrs. | Staff Nurse | Probrs. | |
| A | | | | | | | Sunday<br>Monday<br>Tuesday<br>Wednesday<br>Thursday<br>Friday<br>Saturday |
| B | | | | | | | Sunday<br>Monday<br>Tuesday<br>Wednesday<br>Thursday<br>Friday<br>Saturday |

etc. etc.

and placed on the matron's table for noting day by day any variations which occur. At the bottom of the form space is left for out-patients, theatre, and other special departments, and lastly come the nurses available for extra work, so that at a glance the matron is able to tell exactly whom she has to rely on. To fill in the names of those whose duties have been changed, in the place which they now occupy, is merely a matter of five minutes' work for the matron or her assistant every morning, and with the aid of this clear picture of the disposition of the staff throughout the hospital every day, the more formal registers can be easily made up once a week at an appointed time, without worry about dates or possibility of error. A form of this description adapted to the requirements of the particular hospital could be struck off on any ordinary duplicator at a very trifling cost, and would repay over and over again by the saving of time effected, the trouble involved in its preparation.

It will be seen that a space is left in the Register of Sisters and Nurses for "Subsequent nursing career."

By providing for continuing the record of the nurse after her departure from the hospital, much may be done to spread the influence of the training school, perhaps in remote country villages, perhaps in far distant lands. If nurses know that reports of their doings are welcomed at headquarters they will not be backward to give news of themselves, and though all this "makes work" it has a far-reaching effect for good. In this as in the right performance of all these fastidious details of making and keeping registers, the matron receives as much as she gives. Not the least valuable part of the system which in some institutions elevates the keeping of registers to a fine art, is the educative effect upon the matron herself.

